# RASSF1A Site-Specific Methylation Hotspots in Cancer and Correlation with *RASSF1C* and *MOAP-1*

**DOI:** 10.3390/cancers8060055

**Published:** 2016-06-10

**Authors:** Natalia Volodko, Mohamed Salla, Alaa Zare, El-Arbi Abulghasem, Krista Vincent, Matthew G.K. Benesch, Todd P.W. McMullen, Oliver F. Bathe, Lynne Postovit, Shairaz Baksh

**Affiliations:** 1 Department of Pediatrics, Faculty of Medicine and Dentistry, University of Alberta, 113 Street 87 Avenue, Edmonton, AB T6G 2E1, Canada; volodko@ualberta.ca (N.V.); zare@ualberta.ca (A.Z.); abulghas@ualberta.ca (E.-A.A.); 2 Department of Biochemistry, Faculty of Medicine and Dentistry, University of Alberta, 113 Street 87 Avenue, Edmonton, AB T6G 2E1, Canada; salla@ualberta.ca (M.S.); benesch@ualberta.ca (M.G.K.B.); 3 Department of Experimental Oncology, Faculty of Medicine and Dentistry, University of Alberta, 113 Street 87 Avenue, Edmonton, AB T6G 2E1, Canada; kvincent@ualberta.ca (K.V.); postovit@ualberta.ca (L.P.); 4 Department of Surgical Oncology, Faculty of Medicine and Dentistry, University of Alberta, 113 Street 87 Avenue, Edmonton, AB T6G 2E1, Canada; todd.mcmullen@ualberta.ca; 5 Departments of Surgery, University of Calgary, 1331-29^th^ St NW, Calgary, AB T2N 4N2, Canada; oliver.bathe@albertahealthservices.ca; 6 Tom Baker Cancer Centre, Department of Oncology, University of Calgary, 1331-29^th^ St NW, Calgary, AB T2N 4N2, Canada; 7 Heritage Medical Research Center, Cancer Research Institute of Northern Alberta, University of Alberta, Edmonton, AB T6G 2R7, Canada; 8 Women and Children’s Health Research Institute, Edmonton Clinic Health Academy (ECHA), University of Alberta, 4-081 11405 87 Avenue NW Edmonton, AB T6G 1C9, Canada

**Keywords:** *RASSF1A*, *RASSF1C*, cancer, DNMT, *MOAP-1*, epigenetics

## Abstract

Epigenetic silencing of *RASSF1A* is frequently observed in numerous cancers and has been previously reported. The promoter region of *RASSF1A* is predicted to have 75 CpG sites, and very few studies demonstrate how the methylation of these sites affects expression. In addition, the expression relationship between *RASSF1A* and its downstream target, modulator of apoptosis 1 (MOAP-1), is poorly understood. In this study, we have explored the mRNA expression of *RASSF1A*, *MOAP-1* and the well-characterized splice variant of RASSF1, *RASSF1C*, in cancer cell lines and primary tumors. We confirmed that the *RASSF1A* promoter is robustly methylated within a 32-CpG region in solid tumors and results in lower mRNA expression. The *MOAP-1* promoter contains ~110 CpG sites, but was not found to be methylated in cancer cell lines when 19 predicted CpG sites were explored. Interestingly, *MOAP-1* mRNA expression positively correlated with *RASSF1A* expression in numerous cancers, whereas *RASSF1C* expression remained the same or was increased in cell lines or tissues with epigenetic loss of *RASSF1A*. We speculate that *MOAP-1* and *RASSF1A* may be more intimately connected than originally thought, and the expression of both are warranted in experimental designs exploring the biology of the *RASSF1A/MOAP-1* molecular pathway.

## 1. Introduction

The RASSF family has varied functions and is composed of 10 family members. The expression of most of these family members is controlled by promoter-specific methylation to varying degrees to suggest a tumor suppressor function and importance in growth control. *RASSF1A* and *RASSF1C* originate from the same genomic area on chromosome 3 by alternate splicing using different promoters. *RASSF1A* promoter methylation gene silencing occurs in several solid cancers, and undetectable or low percent methylation is observed in hematological cancers (with the exception of Hodgkin’s lymphoma) [1]. In addition to direct and inflammation driven epigenetic mechanisms regulating *RASSF1A* expression, p53-directed DNMT1 methylation of *RASSF1A* [2], as well as microRNA regulation of *RASSF1A* have been documented [3]. For some patients with solid cancers, epigenetic changes in *RASSF1A* can be detected in leukocytes [4], urine [5], nipple aspirates [6] and saliva [7] to support the identification of circulating tumor cells and to highlight non-invasive methods to detect hypermethylation of *RASSF1A*. Recently, *RASSF1A* hypermethylation was detected in leukocytes in workers exposed to radiation during the Chernobyl Nuclear Power Plant disaster in Russia in 1986 [8] to suggest a high susceptibility of the *RASSF1A* promoter to epigenetic modifications.

RASSF1A is a bona fide tumor suppressor protein that can promote death receptor-dependent cell death via TNF-R1, TRAIL or Fas activation [3,9]. It can associate with the microtubule network, regulate the activity of the anaphase-promoting complex/cyclosome (APC/C)-cdc20 complex/degradation of A and B cyclins [10,11,12] and associate with centromeric γ-tubulin to allow sister chromatid segregation. If *RASSF1A* is absent, improper sister chromatid separation ensues leading to inheritable aneuploidy and malignancy. We have demonstrated that *RASSF1A* can restrict NFκB activation and prevent uncontrolled inflammation in intestinal cells [13]. These biological functions are lost once epigenetic regulation of *RASSF1A* occurs.

Current *Rassf1a* single or double knockout mice generated by various laboratories are viable and fertile. However, by 12–16 months of age, *Rassf1a^−/−^* mice have increased tumor incidence (especially in the breast, lung, gastrointestinal tract and immune system, e.g., B-cell-related lymphomas) and develop tumors in response to chemical carcinogens [14,15]. Beyond six months, we have observed the spontaneous colitis-like phenotype in *Rassf1a^−/−^* mice that was accompanied with increased cytokine production [16] indicating a possible role for RASSF1A in regulating inflammation. *Rassf1a^−/−^Apc*^+/Min^ [17] mice have obstructive polyp formation, and *Rassf1a^−/−^p53^−/−^* reveal decreased survival from >600 days for the *Rassf1a^−/−^* single knockout to <136 days for *Rassf1^−/−^p53^−/−^*, mainly getting sick from the malignancies that develop [18].

Aiding in the ability of *RASSF1A* to promote extrinsic cell death is its downstream effector, MOAP-1. MOAP-1 can also promote intrinsic cell death [9,19], activation of BH3-containig proteins and is regulated in cancer [20] by ubiquitin-dependent degradation. Although the CpG island of *MOAP-1* is 954 base pair long containing about 110 CpG sites within the promoter region (as obtained via MethPrimer [21]), it does not appear to be regulated by promotor-specific methylation in cancers [22] [23]. Since *RASSF1A* is involved in cell death [9], cell cycle control [24,25] and regulation of NFκB [13], the biology of *RASSF1A* appears to suggest that MOAP-1 and RASSF1A may be more linked than originally thought to suggest an overlap of function. In this study, we wanted to explore detailed CpG methylation of *RASSF1A* and link it to *RASSF1C* and *MOAP-1* expression.

## 2. Results

**Figure 1:**
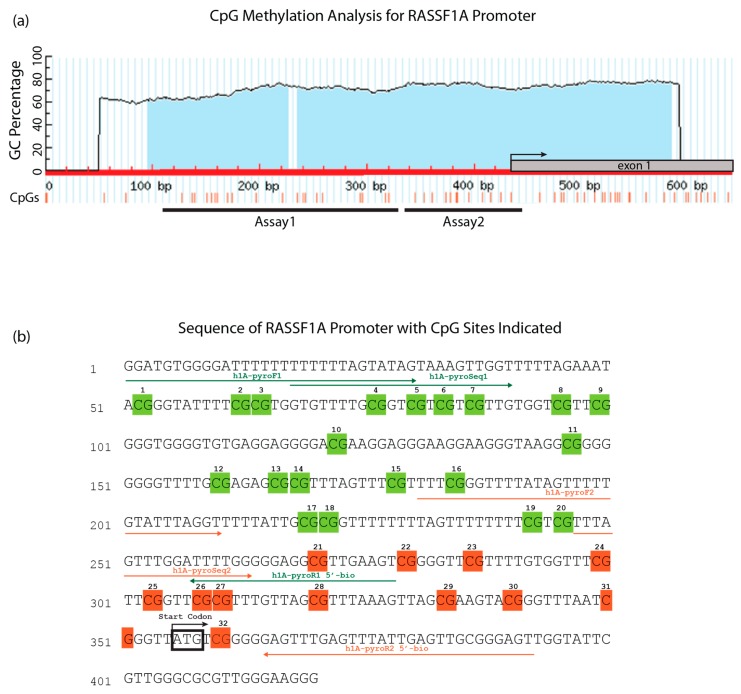
*RASSF1A* CpG island map and the PyroMark assays. (**a**) Part of the *RASSF1A* CpG island, as predicted by MethPrimer [21]. CpG sites are indicated by red strikes. Pyrosequencing assay coverage is shown as black lines. The start codon is indicated by an arrow. (**b**) *RASSF1A* bisulfite-modified DNA sequence with the studied CpGs and primer locations. The start codon is indicated by a black rectangle. Twenty CpGs interrogated by Assay 1 are highlighted in green; the 12 CpG of Assay 2 are highlighted in orange.

**Figure 2:**
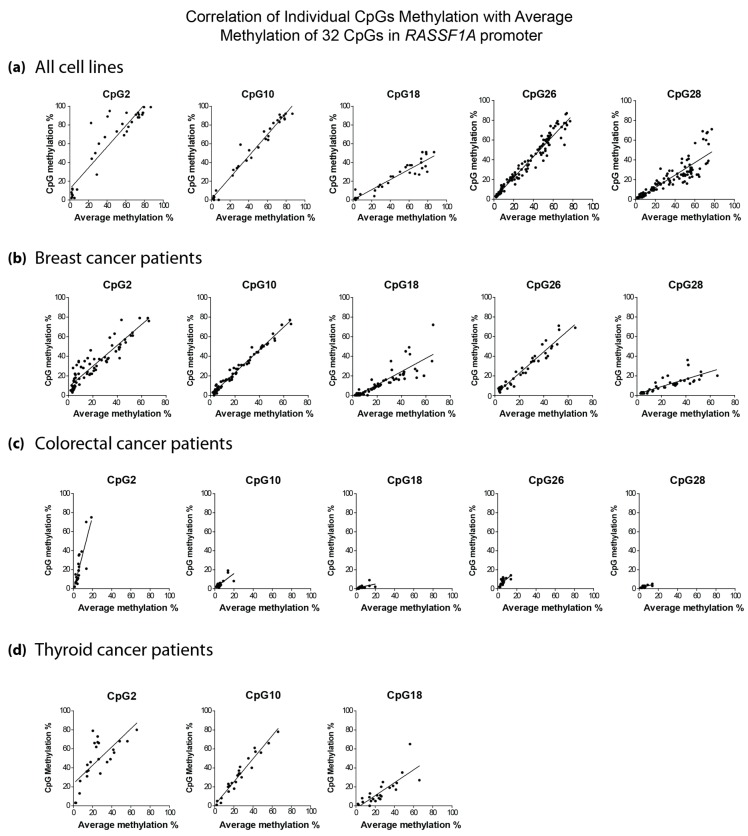
Correlation analysis of *RASSF1A* promoter methylation at the individual CpG site with the average methylation percentage of 32 CpGs in cell lines; *n* = 34 for CpG1, 10 and 18; *n* = 125 for CpG26 and 28 (**a**), breast cancer, *n* = 73 (**b**), colorectal cancer, *n* = 31 (**c**) and thyroid cancer, *n* = 24 (**d**) patients. The x-axis indicates the average methylation of 32 CpGs in samples. The y-axis indicates the individual CpG methylation percentage measured by pyrosequencing. Methylation of all CpGs correlated well with average methylation (r^2^ values ranged between 0.7926 for CpG28 and 0.9939 for CpG9 for cell lines; and between 0.5777 for CpG27 and 0.9886 for Cpg7; all *p*-values are <0.0001). For other CpGs, please see Figure S1a–d.

*RASSF1A* epigenetic silencing has been documented in numerous reports. The frequently-used methylation-specific PCR (MSP) or combined bisulfite modification restriction enzyme analysis (COBRA) techniques can only detect methylation of a few sites, are not quantitative and only give average methylation readout. Here, we developed two pyrosequencing assays covering 32CpGs in the *RASSF1A* promoter (Figure 1). The methylation at individual CpGs correlated with the average methylation percentage, although there was some variation in the methylation percentage of each CpG (Figure 2 and Figure S1a–d). This observation was consistent in cancer cell lines (Figure 2a) and tumor tissues from breast (Figure 2b), colorectal (Figure 2c) and thyroid cancer (Figure 2d). For colorectal cancer, a methylation hotspot was identified whereby CpG 1–7 contributed to most of the methylation observed in the RASSF1A promoter from this patient population. In contrast, the average promoter methylation value for RASSF1A in either breast or thyroid cancer patients can be obtained from methylation % from any of the CpG sites within the RASSF1A promoter. We defined a hotspot as a region with a relatively high methylation in comparison to its surroundings that is found in most samples analyzed.

### 2.1. RASSF1A Promoter Methylation in Cancer Tumor Tissue

We analyzed the *RASSF1A* methylation status in 69 breast cancer patients and 12 reduction mammoplasty controls from cancer-free women. Breast cancer patients were grouped according to tumor feature, invasive and noninvasive, hormone receptor status as Luminal A (Her2−, ER+, PR+/–), Luminal B (Her2+, ER+, PR+/−), Her2 overexpressed (Her2+, ER−, PR–), triple negative (Her2−, ER−, PR−) and, lastly, according to inflammatory breast cancer (IBC) −/+ Her2/Neu. In normal breast reduction surgery tissue the average *RASSF1A* methylation was 7% ± 2.5% *versus* 26% ± 16.6% inbreast cancer tumor tissue. This is close to the average methylation reported in 238 breast cancer patients from Sweden (30.92% ± 17.34 %) [26]. The percentage of hypermethylated samples varied between breast cancer groups. Carcinoma *in situ* *RASSF1A* average methylation was 17% ± 12.5% *versus* invasive breast cancer 27.4% ± 17%, confirming previous reports that *RASSF1A* methylation may be associated with the degree of cancer invasion and be an early event in breast cancer. 

**Figure 3:**
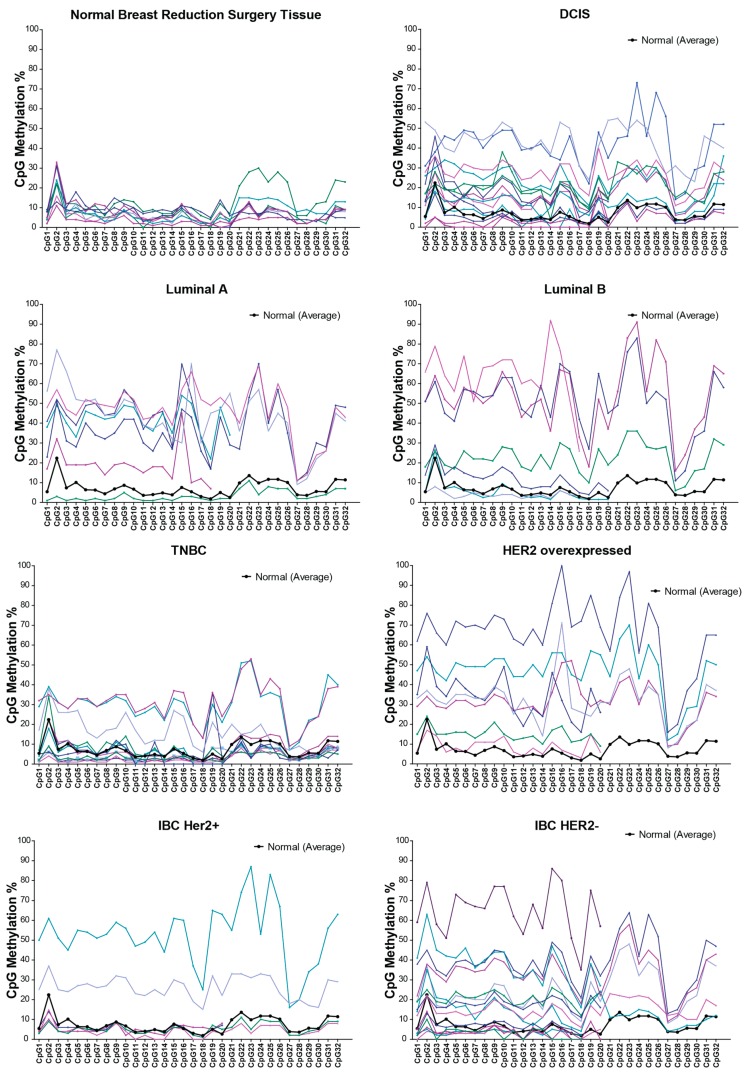
*RASSF1A* promoter methylation percentage of individual CpGs sites in breast cancer patients, *n* = 73. The individual CpG methylation percentage indicates what percentage of DNA molecules is methylated at this site in the sample. Although the methylation pattern is different in different types of breast cancer, CpG2, 9, 13–14, 16, 19, 26 and 31 are the methylation hotspots in all types of breast cancer, suggesting a conserved methylation mechanism. IBC, inflammatory breast cancer; DCIS, ductal carcinoma *in situ*; TNBC, triple-negative breast cancer.

Methylation of *RASSF1A* showed significant differences between breast cancers when grouped according to hormone receptor status. In Luminal A (Her2−), *RASSF1A* average methylation was 33% ± 16%, whereas in Luminal B (Her2+), *RASSF1A* average methylation was 44% ± 22%, which suggested that the Her2/Neu receptor may have a role in the increase seen in *RASSF1A* methylation. This was also supported by the results of *RASSF1A* average methylation in Her2/Neu overexpressed tumors (38% ± 17%) and the *RASSF1A* average methylation seen in triple-negative breast cancer (TNBC) (10% ± 9.5%; please see Figure 3). Similar findings were also seen in tumors from IBC patients with varying receptor status. In IBC, *RASSF1A* average methylation was higher in the IBC/Her2+ subtypes at 39.3% ± 22% and lower in the IBC/Her2− subtypes at 16% ± 5%. We speculate that averaging *RASSF1A* methylation of all 32 CpG areas may overlook the areas that are considered to be hot spots for methylation (see Figure 3). For breast cancer, these areas map to CpG2, 9, 13, 14–16, 19, 23–25 and 31, suggesting common susceptible CpGs regardless of the tumor feature, hormone receptor and inflammation status of breast cancer.

**Figure 4:**
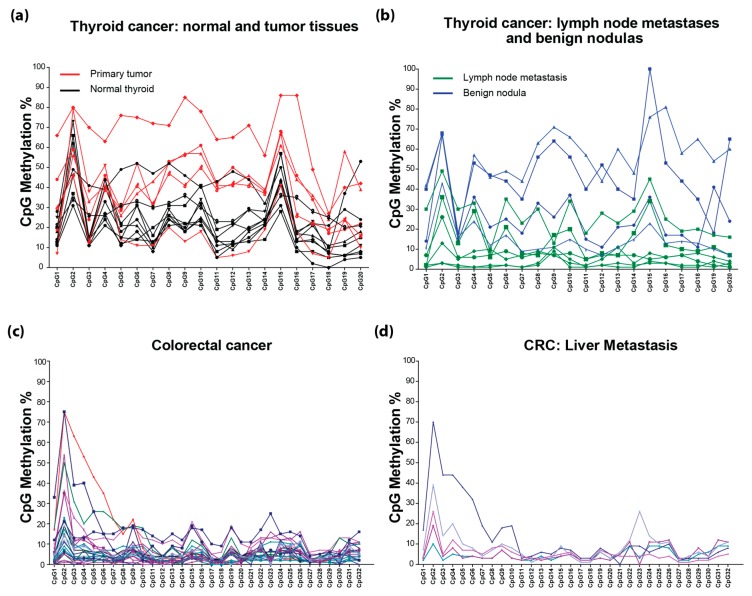
The methylation percentage of the individual CpGs in the *RASSF1A* promoter in thyroid cancer patients, *n* = 24 (**a**–**c**) colorectal cancer patients, *n* = 26; (**d**) colorectal cancer liver metastasis, *n* = 5. The individual CpG methylation percentage indicates what percentage of DNA molecules are methylated at this site in the sample.

In thyroid cancer, *RASSF1A* methylation has been studied mainly by MSP, and a recent meta-analysis based on 11 studies suggested that it may play a role in papillary thyroid cancer [27]. To our knowledge, this is the first attempt to look at the detailed CpG methylation in thyroid cancer. Analysis of 24 thyroid cancer patient tumors revealed that *RASSF1A* methylation was higher in primary tumors compared to normal thyroid tissues, and methylation peaks and troughs were similar to breast cancer tumors with peaks at CpG2, 9 and 14–16 and troughs at CpG3, 11–14 and 18 (Figure 4a). Surprisingly, lymph node metastatic tumors from thyroid cancer patients revealed a lower methylation than the primary tumor for unknown reasons (Figure 4b, *n* = 5 for lymph node metastatic tumors).

*RASSF1A* methylation in colorectal cancers has been studied extensively with the percentage of patients with hypermethylated *RASSF1A* ranging from 0% [28] to 81% [29]. Individual CpG methylation analysis of 27 colorectal cancer patients revealed a unique pattern of *RASSF1A* methylation with a hotspot at CpG 1–7 showing robust methylation >30% (Figure 4c). This is in contrast to what is observed in breast and thyroid cancers, where methylation appears >30% throughout the 32 CpGs (Figure 3 and Figure 4a,b). If RASSF1A methylation analysis design in CRC patients does not include CpG sites 1–7, then average methylation of <30% will be detected in CRC tumor tissues and be misleading. The importance of CpG sites 1–7 is also observed for metastatic lesions in the liver in CRC patients to suggest that the metastatic lesions resemble the primary tumors, in contrast to what we can observed for metastatic sites for thyroid cancer patients (compare Figure 4d to Figure 4b). This surprising hotspot for methylation in CRC is potentially one explanation for the varied reports of *RASSF1A* methylation in CRC samples. Analysis of more CRC samples is needed to explore why CRC reveals a localized hotspot for the methylation of the *RASSF1A* promoter. In addition, we are also currently exploring why lymph node metastasis in thyroid cancer patients does not harbor significant levels of *RASSF1A* promoter-specific methylation (Figure 4b). 

### 2.2. RASSF1A Promoter Methylation in Normal and Cancer Cell Lines

**Figure 5:**
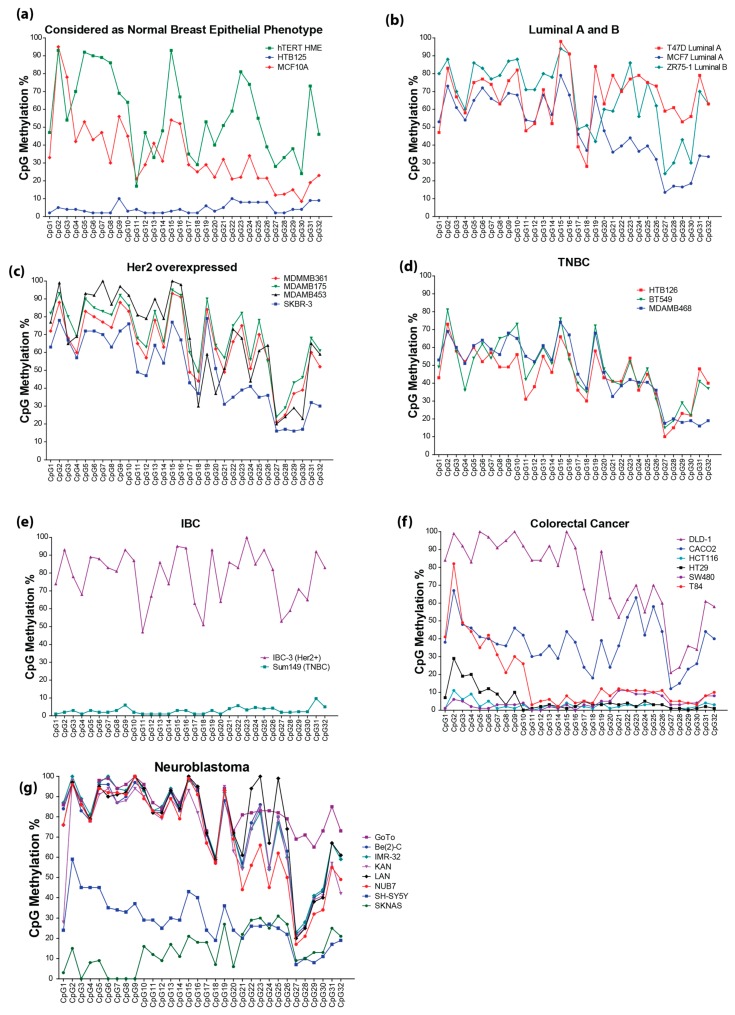
*RASSF1A* promoter methylation percentage of individual CpGs sites in cell lines. (**a**) Breast epithelial cell lines considered as normal. (**b**) Luminal A and Luminal B breast cancer. (**c**) Her2 overexpressed breast cancer. (**d**) Triple-negative breast cancer (TNBC). (**e**) Inflammatory breast cancer (IBC). (**f**) Colorectal cancer. (**g**) Neuroblastoma. The individual CpG methylation percentage indicates what percentage of DNA molecules is methylated at this site in the sample.

Analysis of 15 breast cancer cell lines revealed significant variations amongst them. MCF10A and hTERT HME (human telomerase reverse transcriptase stably transfected human mammary epithelial cell line) are generally considered and utilized as “normal” cell lines in breast cancer research. However, the average methylation of *RASSF1A* in both cell lines was found to be significantly higher than the HTB125 normal breast epithelial cell line or methylation found in tissues from breast reduction surgery patients (Figure 5a). We have detected about 4.5% average methylation of *RASSF1A* in HTB125 normal breast epithelial cells, while MCF10A had >30% and hTERT >50% average methylation of *RASSF1A* promoter. This result suggests that these cells should not be utilized as normal cells with respect to *RASSF1A* expression and would most likely have methylation of other highly susceptible genes, such as p16, DAPK and caspase 8, that normally have expression loss when the *RASSF1A* promoter is methylated [3,30]. Caution is required when exploring RASSF1A function in any of these cell lines due to this inherent variability. 

Methylation of *RASSF1A* demonstrated significant differences between breast cancer cell lines according to hormone receptor status. In Luminal A (Figure 5b, MCF7 and T47D), *RASSF1A* average methylation is 58% ± 16.9%, whereas in Luminal B (Figure 5b, ZR-75), *RASSF1A* average methylation is 67% ± 18.9%, which again suggests that the Her2 receptor may have a role in the increase seen in *RASSF1A* methylation. This was also supported by the results of *RASSF1A* average methylation in Her2 overexpressed cell lines (Figure 5c; MDA-MB-453, MDA-MB-175, MDA-MB-361 and SKBR-3) at 63% ± 20.6% *versus* TNBC cells (Figure 5d; Her2−: HTB 126, MDA-MB-468 and BT549) that had *RASSF1A* average methylation at 46.5% ± 16%. Similarly, IBC *RASSF1A* average methylation was higher in the IBC/Her2 overexpressed subtypes represented with IBC-3 at 79% ± 14% and lower in the IBC/Her2 triple negative subtype, Sum149, 2.8% ± 1.9% (Figure 5e). This significant difference in *RASSF1A* average methylation between Her2 positive and negative subtypes suggests that the lack of *RASSF1A* expression may have a role in the expression status of Her2, thus influencing the biology of the breast cancer cell. Interestingly, similar patterns of CpG *RASSF1A* promoter methylation hotspots in breast cancer tissue are found in cell lines, including CpG2, 9, 13, 14–16, 19, 23–25 and 31, except for CpG5-7, which was highly methylated only in cell lines. Similar to breast cancer tissues, these methylation hotspots within the *RASSF1A* promoter in breast cancer cell lines may depend on hormone receptor status and stage of disease.

Similarly, in colorectal cancer cell lines, *RASSF1A* epigenetic silencing varied with DLD-1 (Dukes’ type C colorectal adenocarcinoma with chromosomal and microsatellite instability and harboring mutations in K-Ras, p53 and PI3KC (P-I3-kinase), while wild-type for BRaf and PTEN [31]) and CaCO-2 having somewhat similar variation in individual CpG methylation. T84 robustly demonstrated methylation of CpG sites 1–7 similar to what is observed in CRC primary tissues (Figure 5f). CaCO-2 had robust methylation of CpG sites 1–3 and 21–27, while DLD-1 had robust methylation of almost all of the CpGs. SW480 only has mutations in K-Ras and p53. HCT116 cells have mutations in K-Ras and PI3KC. HT-29 cells have mutations in K-Ras, BRaf, PI3KC and p53. CaCO-2 cells have mutations in p53. Thus, overall, methylation of *RASSF1A* on CpG 1–7 and the presence of K-Ras mutations may define an aggressive CRC cell (Figure 4b and Figure 5f). Neuroblastoma cell lines also had robust methylation of almost all of the CpGs (Figure 5g) and can harbor mutations in both K-Ras and B-Raf [32]. These results demonstrate the importance of mapping individual CpGs for different cancers. Average *RASSF1A* methylation masks the importance of CpG hotspots, as can be observed in Figure 4c for CRC (Figure 4c).

**Figure 6:**
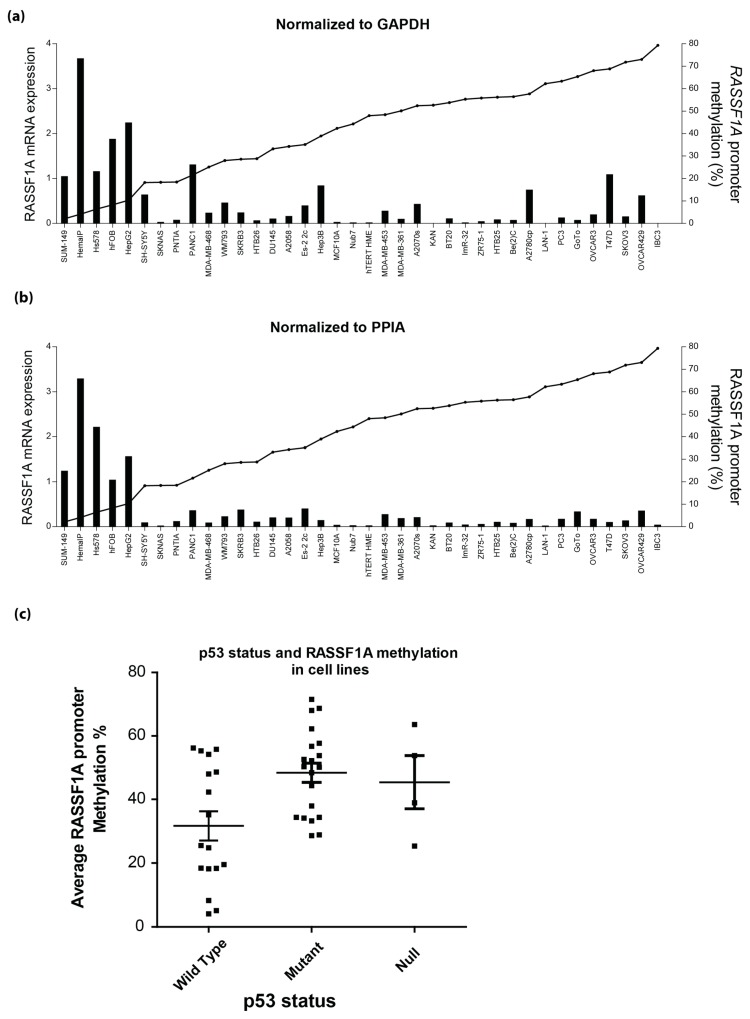
*RASSF1A* expression and methylation in cell lines. (**a**,**b**) *RASSF1A* methylation results in its mRNA expression silencing in cell lines. GAPDH mRNA expression (**a**) was used to normalize *RASSF1A* expression, and similar results are obtained if using peptidylprolyl isomerase A as a reference gene (**b**). (**c**) p53 status and *RASSF1A* methylation in cell lines (*n* = 20 for all except for p53^Null^ cells, where *n* = 4). Cell lines were separated into groups according to their p53 status and average *RASSF1A* methylation plotted.

A summary of *RASSF1A* methylation status *versus* *RASSF1A* mRNA expression in numerous cancer cell lines suggests that >10%–20% *RASSF1A* methylation may result in significant loss of *RASSF1A* expression (Figure 6a,b). Similarly, a subgroup of colon cancer cells supported the results in Figure 6a,b to suggest that RASSF1A methylation >20%–30% would result in a significant loss of RASSF1A mRNA expression (Figure S3). In addition, we found that higher *RASSF1A* methylation was found in the context of p53 mutant and p53 null cells (48% ± 4% and 45% ± 4%, respectively) *vs.* the 31% ± 4% found in p53 wild-type cells (Figure 6b). It is believed that RASSF1A epigenetic loss is an early event during tumorigenesis that may represent an early driver of malignancy. Loss of p53 may follow the loss of *RASSF1A* in numerous cancers. Although this is a limited number of cells, it does illustrate that the p53 status may be important to document in the context of the loss of *RASSF1A.*

### 2.3. mRNA Expression Correlation of DNMT1and DNMT3B with RASSF1A

**Figure 7:**
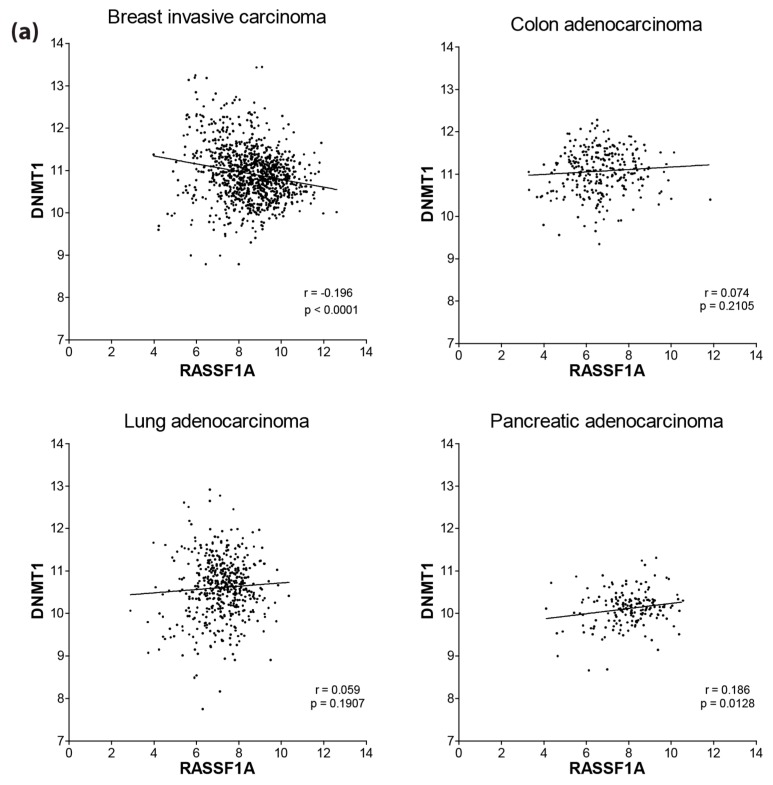

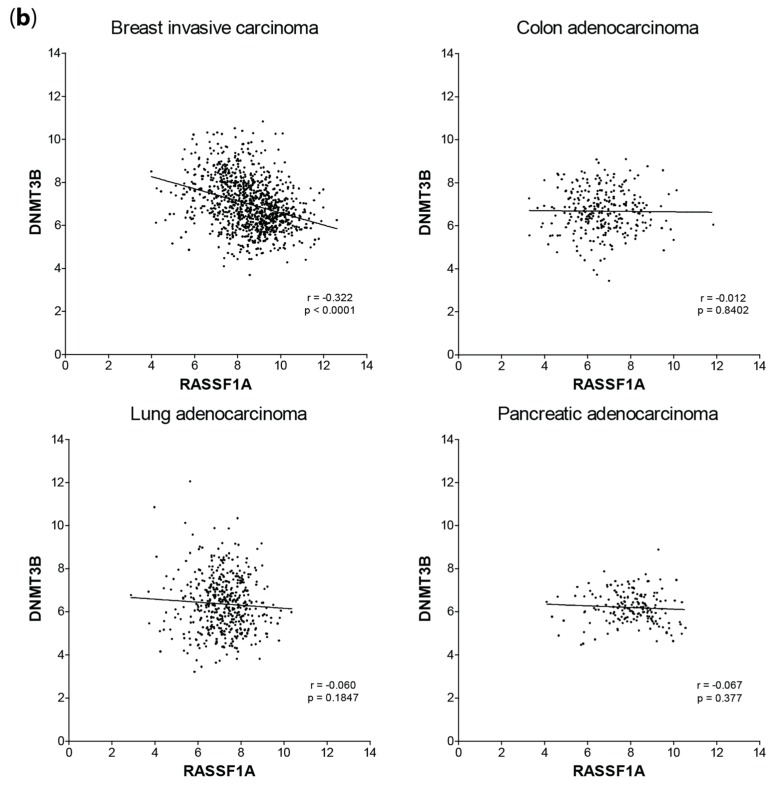
Correlation of RASSF1A expression with DNMT1 and DNMT3B expression in patient tumors from different cancers. Scatterplot of the expression values (*log*_2_[RSEM+1]) of RASSF1A (horizontal) and (**a**) DNMT1 or (**b**) DNMT3B (vertical) for primary tumors from TCGA (The Cancer Genome Atlas) breast cancer (*n* = 1062), colorectal adenocarcinoma (*n* = 286), lung adenocarcinoma (*n* = 488) and pancreatic adenocarcinoma (*n* = 178) patient samples. Lines indicate linear regression; Spearman’s correlation coefficients and associated *p*-values are displayed in the bottom right corners.

*RASSF1A* is epigenetically silenced by promoter-specific methylation. There is evidence for the role of DNMT1/3B in regulating the methylation of the *RASSF1A* promoter [2]. We therefore explored the expression status of *DNMT1* and *DNMT*B *versus* *RASSF1A* expression in various cancers utilizing data from The Cancer Genome Atlas (TGCA) (Figure 7). Although Spearman’s r was not high, there appears to be a negative correlation of *DNMT1* or *DNMT3B* mRNA expression with *RASSF1A* expression only in breast cancer (Figure 7a,b). Although a surprising observation, these data may suggest that in many cancers (apart from breast cancer), the methyltransferase activity changes of DNMT (as opposed to an mRNA changes) may govern its ability to modulate epigenetic changes.

### 2.4. mRNA Expression Correlation of MOAP-1 with RASSF1A

**Figure 8:**
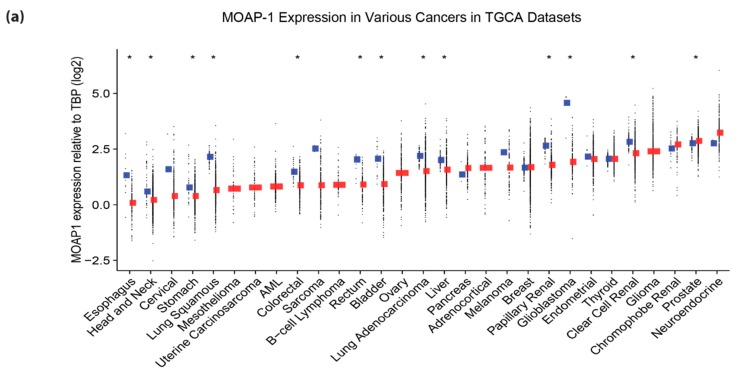

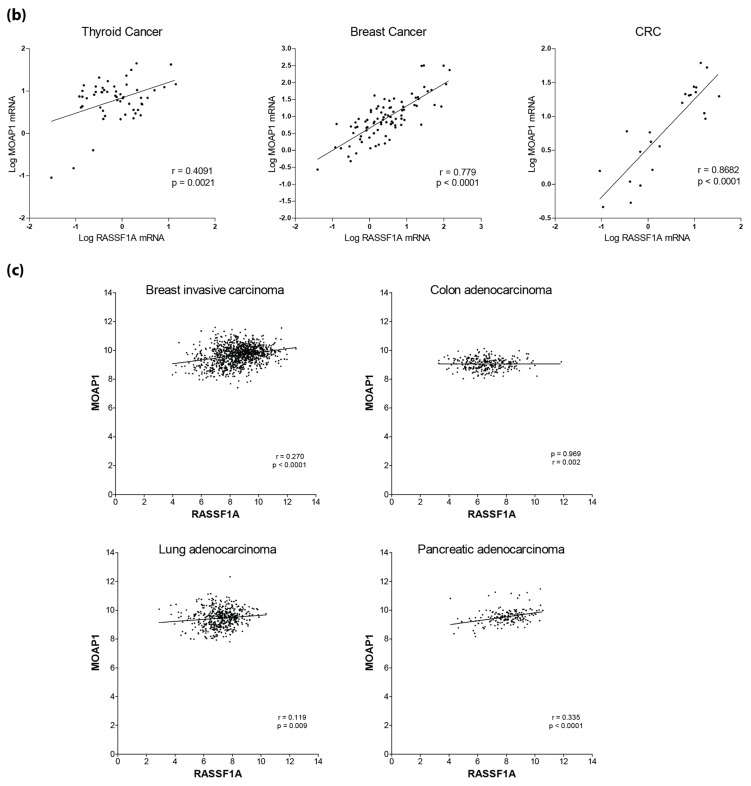
*MOAP-1* expression and its correlation with *RASSF1A* expression in different cancers. (**a**) *MOAP-1* expression in different cancers (red) compared to normal (blue). TPB, TATA-binding protein. (**b**) Scatterplot of the expression values (normalized to *GAPDH*) of *RASSF1A* and *MOAP-1* for thyroid (*n* = 55), breast (*n* = 77) and colorectal cancer (*n* = 31) patients. (**c**) Scatterplot of the expression values (log2[RSEM+1]) of *RASSF1A* (horizontal) and *MOAP*-*1* (vertical) for primary tumors from TCGA breast cancer (*n* = 1062), colorectal adenocarcinoma (*n* = 286), lung adenocarcinoma (*n* = 488) and pancreatic adenocarcinoma (n = 178), patient samples. In (b,c), lines indicate linear regression; Spearman’s correlation coefficients and associated *p*-values are displayed in the bottom right corners.

Recently, we published that the pro-apoptotic protein, MOAP-1, and downstream effector of the *RASSF1A* pro-apoptotic pathway is a tumor suppressor protein whose expression varies widely in cancers [20]. Analysis of data obtained from TCGA reveals evidence for the reduction of MOAP-1 expression in numerous cancers with the lung, cervical, colorectal, rectal, bladder cancers and glioblastoma (Figure 8, red tagged data points). The majority of those cancers also have epigenetic loss of *RASSF1A*. This is somewhat surprising considering that there is strong evidence for MOAP-1 expression being controlled by post-translational ubiquitination mechanisms utilizing several E3 ligases with a half-life of <30 min [19,33,34]. That said, the *MOAP-1* promoter contains ~120 CpGs that potentially could be methylated (please see Figure S2). Empirical testing of 19 of these sites using the PyroMark technology on colon cancer cells reveals <5% methylation of these sites. Furthermore, these 19 CpG sites can only be methylated to <25% with SssI methyltransferase to suggest a very low susceptibility (or high resistance) to become methylated [22]. In contrast, most of the cells from lung, cervical, colorectal, rectal, bladder cancers and glioblastoma tumors have *RASSF1A* methylation to >50% [3], and SssI methyltransferase can methylate colon cancer cells to >90% [22] to suggest robust DNA methylation susceptibility mechanisms within the 32 CpG sites on the *RASSF1A* promoter. Thus, if expression changes in MOAP-1 do occur, it may be through the remaining ~100 CpGs that potentially could be methylated or by non-DNA methylation mechanisms, such as transcriptional regulation by MaFB, NFκB and/or STAT1, as described by Law *et al.* [19] or by microRNA regulation, as described by Volodko *et al.* [3].

According to the GeneCards databse [35], 31 microRNAs are predicted to target MOAP-1, including several that target *RASSF1A.* It has been demonstrated that mir-1228 [36] and mir-25 in lung cancer [37] can regulate the expression levels of MOAP-1. Both p53 and MOAP-1 appear to be targets of mir-25 and mir-1228, facilitating the progression of cancer cells in the cell cycle [36,38]. Transfecting A549 (non-small cell lung cancer) and 95-D (lung carcinoma) cells with a miR-25 inhibitor was shown to favor an apoptotic morphology [37]. The TRAIL death receptor-4 (DR-4) has also been identified as a potential mir-25 target, with reports of mir-25 being upregulated in prostate carcinoma, gastric adenocarcinoma, cholangiocarcinoma and other human cancers [39]. Dysregulation of mir-1228 has also been shown in many tumors, such as lung adenocarcinoma and breast cancer [36]. Interestingly, the anti-tumor drug resveratrol reduces the expression of mir-1228 in human non-small lung cancer [40].

As mentioned earlier, several biological functions of MOAP-1 and RASSF1A appear to overlap, and MOAP-1 is a key effector for RASSF1A biology [20]. We explored the mRNA expression of both RASSF1A and MOAP-1 in cancers. Correlation plots were quite revealing in empirical analysis using qRT-PCR in cancer tissues (Figure 8b). Data analysis from TCGA suggests that changes in MOAP-1 and RASSF1A do not occur in a random fashion in breast, lung and pancreatic cancers that have a *p*-value of <0.05. However, the correlation coefficients suggest that expression levels of MOAP-1 and RASSF1A may influence each other, but the correlation may not be as simple to interpret as the results from the empirical testing (Figure 8c). Thus, it is quite important to know both the *MOAP-1* and *RASSF1A* status of cell lines/tissues utilized to explore the biology of RASSF1A or any other RASSF family member associated with MOAP-1.

### 2.5. mRNA Expression Correlation of RASSF1C with RASSF1A

The RASSF1 gene has eight transcripts (A–H) arising from alternative splicing and differential promoter activity [3]. Among the RASSF1 subtypes, *RASSF1A* and *RASSF1C* are the most extensively studied members that have been demonstrated to be localized to microtubules and involved in growth control. However, growing evidence suggests a tumor suppressor function for *RASSF1A* and an oncogenic function for *RASSF1C*, especially in breast and lung cancer [41,42]. Thus, despite harboring 60% amino acid identity (mainly after amino acid 121 of *RASSF1A*), *RASSF1A* and *RASSF1C* display distinctive biological properties [3]. Many of the RASSF1 family members can homodimerize and heterodimerize with each other, including a RASSF1A/1C complex. Recently, an intriguing mechanism was proposed to suggest that the loss of RASSF1A would trigger either the release of RASSF1C from a RASSF1A/1C complex or promote upregulation of mRNA for *RASSF1A.* The result of either mechanism would be an increased pool of unbound RASSF1C to result in the activation of Src kinases and transcriptional activation of the YES associated protein (YAP) to modulate proliferation. Vlahov *et al.* demonstrated that protein-protein interactions of both RASSF1A and RASSF1C revealed binding of both isoforms to the tyrosine kinases c-Src, FYN and YES, but a unique association of RASSF1A to CSK, a SRC inhibitory kinase that phosphorylates SRC kinases at Y527 [43]. The loss of RASSF1A releases CSK and allows RASSF1C-induced activation of SRC kinases [43].

**Figure 9:**
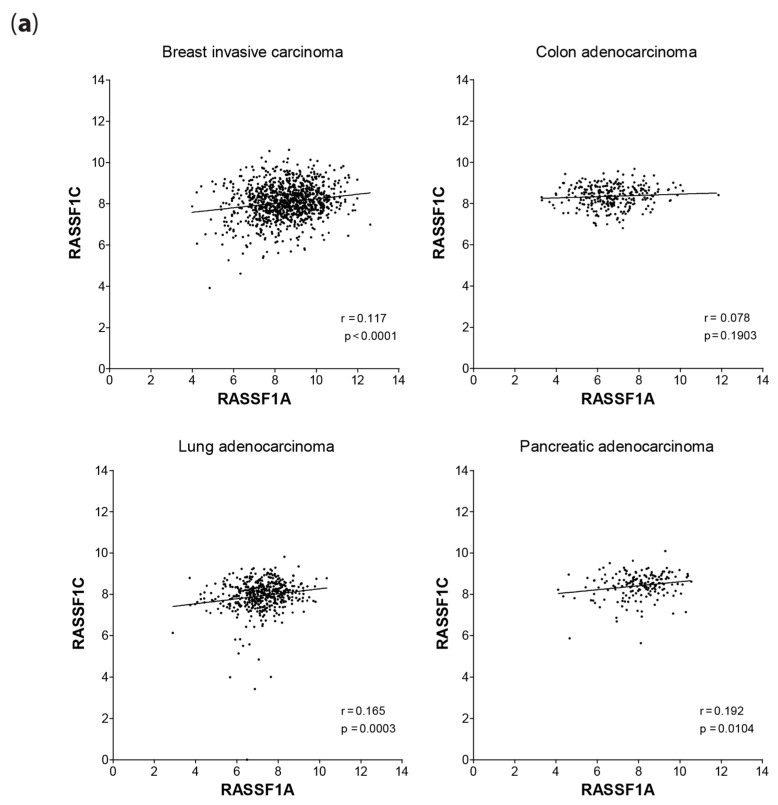

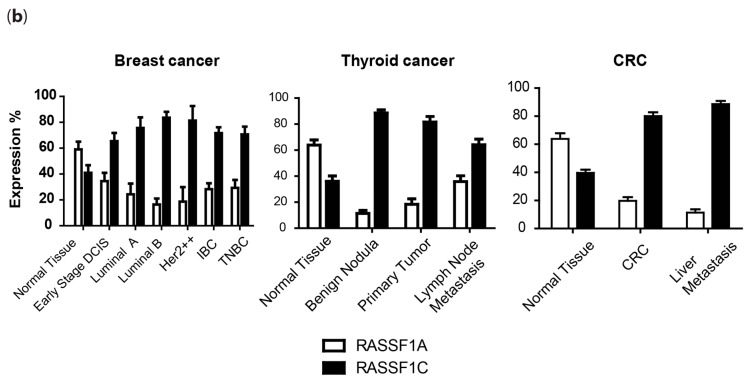
Correlation of *RASSF1A* expression with *RASSF1C* expression in cancers. (**a**) Scatterplot of the expression values (*log*_2_[RSEM+1]) of RASSF1A and RASSF1C for primary tumors from TCGA breast cancer (*n* = 1062), colorectal adenocarcinoma (*n* = 286), lung adenocarcinoma (*n* = 488) and pancreatic adenocarcinoma (*n* = 178) patient samples. Lines indicate linear regression; Spearman’s correlation coefficients and associated *p*-values are displayed in the bottom right corners. (**b**) In breast, thyroid and colon cancer patients, *RASSF1A* and *RASSF1C* expressions were summed and the percentage of *RASSF1A* and *RASSF1C* expression calculated for each patient. Mean ± SD percentages are presented. For most breast cancers subtypes, *n* = 11–21; for thyroid cancer subtypes, *n* = 8–13; for colorectal cancers subtypes, CRC, n = 26; and liver metastasis, *n* = 5. Normal epithelial tissue was obtained from breast reduction surgery patients (*n* = 13) and used as normal tissue values for all three cancers. Expression of 1A has been shown on The Protein Atlas [44] and Genecards [45] databases to be comparable in these three tissue types.

Based on this observation, we explored mRNA expression changes in both *RASSF1A* and *RASSF1C* in cancers from TCGA (Figure 9a). Similar to MOAP-1, data analysis from TCGA suggests that changes in *RASSF1C* and *RASSF1A* do not occur in a random fashion in breast, lung and pancreatic cancers that have a *p*-value of < 0.05. However, correlation coefficients reveal poor linear changes in the expression of RASSF1A with RASSF1C. That said, empirical testing of mRNA levels of *RASSF1A* and *RASSF1C* by qRT-PCR in breast cancer tumor tissues (Figure 9b) revealed that expression changes of *RASSF1C* can occur as a result of the loss of *RASSF1A*. It can be seen that RASSF1A expression decreases and RASSF1C increases in cancers compared to normal. Empirical testing of *RASSF1A:RASSF1C* mRNA levels in thyroid and colorectal tumor tissues support our observations in breast cancer. Furthermore, the selection pressure towards RASSF1A/RASSF1C changes in cancer is sustained in metastatic sites for CRC (liver), but not for thyroid (lymph node; Figure 9b). We are currently exploring why lymph node metastasis as a result of thyroid cancers does not harbor RASSF1A promoter-specific methylation.

Nevertheless, Figure 9b supports the observation by Vlahov *et al.* [43] to suggest that RASSF1A/RASSF1C expression levels can be altered in many cancers to result in the activation of Src [43]. These observations add to the need to monitor the expression of both *RASSF1A* and *RASSF1C* (and Src activity) when exploring the new and established biology of *RASSF1A*. Database search results for RASSF1 cannot be equated to RASSF1A unless the methodology has two probes for RASSF1A and RASSF1C (such as RNA-sequencing) to avoid underestimating the expression loss of *RASSF1A.*

## 3. Discussion

*RASSF1A* methylation analysis revealed some interesting aspects of the regulation of *RASSF1A* in cancers. It has been previously shown by MSP and COBRA that some CpGs in the *RASSF1A* promoter are more relevant biologically for transcription regulation. In lung cancer, it was found that there are eight CpGs in the promoter area and six in the first exon that transcriptionally are more important than the other CpGs [46]. In breast cancer, it also has been shown that *RASSF1A* methylation has a progressive nature starting in the first exon and spreading into the promoter and promoter methylation (not exon 1 methylation) correlated with *RASSF1A* expression silencing [47]. However, the extent of methylation at individual CpG sites remains largely unknown. We utilized pyrosequencing to explore the methylation signature of 32 of 75 potential CpGs in the promoter of the *RASSF1A* gene in both cell lines and tumor tissues. This analysis revealed CpG methylation hotspots within the *RASSF1A* promoter in both patient tumor tissues and cancer cell lines. Although, *RASSF1A* is heavily methylated, hope is there that it can be reversed with the DNA methyltransferase inhibitors, 5-azacytidine or 5-aza-2’-deoxycytidine (Decitabine) [48], or with the adenosine metabolism uncoupler, dipyridamole (DIPY), in combination with a synthetic antifolate activator (3-*O*-(3,4,5-trimethoxybenzoyl)-(2)-epicatechin; TMECG) [49]. Treatment of cells with both resulted in increased cell death and growth inhibition. Active research into how to efficiently deliver 5-azacytidine or 5-aza-2’-deoxycytidine to tumor sites will enhance the usefulness of this DNA methyltransferase inhibitor.

*RASSF1A* expression has been demonstrated to be regulated by epigenetic silencing directly by DNMT [31] and indirectly by p53/death-associated protein 6 (DAXX) biology [2]. It was demonstrated that p53 binding to the *RASSF1A* promoter resulted in the recruitment of DAXX and DNMT1, DNA methylation and inactivation of the *RASSF1A* promoter. Interestingly, it was the DAXX expression levels (and not the p53 expression levels) that affected the rates of *RASSF1A* methylation [2]. Our data in Figure 6c may suggest some influence of p53 on the methylation status of RASSF1A, but further population-based analysis may be needed to confirm the influence of p53 mutational status on RASSF1A promoter-specific methylation. In a genome-wide RNAi screen, it was found that homeobox protein HOXB3 is required for *RASSF1A* promoter hypermethylation [31,50]. It appears that HOXB3 binds to the *DMNT3B* gene to increase its expression. DNMT3B then is recruited to the *RASSF1A* locus through interactions with polycomb repressor complex 2 (PRC2) and MYC, where it methylates the *RASSF1A* promoter [31]. RASSF1A may well be regulated through multiple mechanisms depending on tissue types.

Epigenetic regulation of *RASSF1A* has also been demonstrated to be modulated by long non-coding RNA (lncRNA). It was shown that non-spliced lncRNA transcribed from the antisense strand of *RASSF1A* forms an RNA/DNA hybrid at the *RASSF1A* transcription site and PRC2 to the *RASSF1A* promoter. This results in increased methylation of histone H3K27 at the *RASSF1A* promoter and reduction of transcriptional activity [51]. Also, more than likely, the discrepancies between *RASSF1A* promoter methylation and its mRNA expression level in some samples could be explained by micro-RNA (miRNA) involvement in *RASSF1A* expression. Computer analysis predicts that *RASSF1A* mRNA can be targeted by at least fifteen miRNAs (miR-326, -330, -149, -16, -497, -504, -410, -99a, -99b, -100, -124, -193, -193b, -182, -181a,b,c,d) [3]. Recent empirical study proved that miR-181b targets and regulates the expression of *RASSF1A* in colorectal cancer [52]. mir-602 has also been shown to change *RASSF1A* mRNA expression in hepatoma cells and hepatocellular carcinoma [53]. Therefore, all of these mechanisms may be in play to regulate *RASSF1A* and all warrant detailed investigations in cancer and other diseases that have expression loss of *RASSF1A*.

Interestingly, *MOAP-1* expression positively correlated with *RASSF1A* expression to suggest an intimate connection beyond biological function. Although located on chromosomes 3p and 14q, it will be interesting to explore why there is this connection between these two proteins and genes. Furthermore, since *RASSF1C* expression and/or function are heightened in the context of the loss of *RASSF1A*, it will be interesting to see how MOAP-1 biology is affected by the loss of *RASSF1A*. It is known that death receptor-dependent cell death (or extrinsic cell death) is significantly lost when *RASSF1A* is epigenetically silenced, but it remains to be determined if intrinsic cell death pathways are lost or MOAP-1 stability and ubiquitination status are affected by increased *RASSF1C* expression and loss of *RASSF1A*.

Epigenetic loss of *RASSF1A* has now been patented by several individuals as a diagnostic test for some cancers [54,55] and represents a target for restoring normal biological function to the cell by reversing DNA methylation [56]. Although several groups have successfully restored *RASSF1A* expression levels using 5-azacytidine or 5-aza-2’-deoxycytidine, targeting to specific tissues and specificity for *RASSF1A* are limitations on its clinical applications. Epigenetic regulation by DNA methylation may not be the predominant choice for mRNA regulation in all cancers. Detailed analysis of *RASSF1A* mRNA expression in each cancer is needed to better understand how it is regulated and how to regain function in that tissue/cancer. As observed in this study, *RASSF1A* epigenetic silencing is robust and widespread within the *RASSF1A* promoter in breast, thyroid and neuroblastoma tumor tissues and cell lines, whereas in CRC tumor tissues and cell lines, methylation of *RASSF1A* is not widespread over the 32 CpGs explored in this study. This may suggest that factors beyond the presence of susceptible CpG sites are likely responsible for the level of epigenetic silencing observed in these and other cancers. This study has begun to uncover the role of each CpG in controlling *RASSF1A* expression, and more analysis is needed to understand how DNA methylation, microRNA, long noncoding RNA and other mechanisms (such as histone modifications around the chromosome 3p.21) can cooperatively regulate *RASSF1A* gene expression.

## 4. Materials and Methods

### 4.1. Cancer Samples

The breast cancer tissues were obtained from the Alberta Cancer Research BioBank; colorectal cancer samples were obtained from the University of Calgary Gastrointestinal/Hepatobiliary Tumor; and thyroid cancer samples from Todd P.W. McMullen. All tissues were snap frozen within 30 min to preserve the RNA.

### 4.2. Cell Lines

HCT-116, SW480, T84 and HT29 (Eytan Wine); HemaLP, A2070s, A2780cp, A2058, WM793 (Sujata Persad, University of Alberta, Edmonton, AB, Canada); PNTIA, Hs578, HepG2, Hep3B, DU145, PC3, Hep3B (CRUK, London, UK); SH-SY5Y, SKNAS, Nub7, Be(2)C, A2780cp, LAN-1, ImR-32, GoTo, KAN (Roseline Godbout, University of Alberta, Edmonton, AB, Canada); PANC1, MDAMB-468, SKRB3, HTB26, MCF10A, hTERT HME, MDAMB453, MDAMB361, BT20, ZR75-1, HTB25, T47D (Mary Hitt/David Murray/Bonnie Andrais, University of Alberta, Edmonton, AB, Canada, and Cross Cancer Institute, Edmonton, AB, Canada); OVCAR3, SKOV3, Es-2 2c (YangXin Fu, University of Alberta, AB, Canada); IBC-3 (Wendy Woodward, MD Anderson Cancer Center); and DLD-1. SUM149 was obtained from Sophia Merajver, University of Michigan Medical School and School of Public Health, MI, USA.

### 4.3. DNA Isolation and Bisulfite Treatment

Extraction of genomic DNA from biopsies and cells was performed using the QIAGEN Allprep DNA/RNA Mini Kit (Qiagen, Hilden, Germany) and quantified with a NanoDrop ND-1000 (PeqLab, Erlangen, Germany). Bisulfite modification of genomic DNA (1 μg), converting all unmethylated, but not methylated cytosines to uracil, was performed using the EZ DNA Methylation-Gold kit (Zymo Research, Irvine, CA, USA) with slight modification of the manufacturer’s protocol (DNA was eluted in 30 μL M-elution buffer instead of the 10 μL recommended). The bisulfite-treated DNA was used for pyrosequencing. The efficiency of the bisulfite conversion was checked by pyrosequencing.

### 4.4. Methylation Analysis by Pyrosequencing

Pyrosequencing [57] was performed on a PyroMark Q24 system (Qiagen) with 2 sets of primers (sequences are provided in Table 1). The Assay2 covers 11 CpGs in the promoter and 1 CpG in exon 1 of the *RASSF1A.* Assay1 covers 20 CpGs located right upstream of the 12 CpGs covered by Assay2 (Figure 1). The PCR was performed using PyroMark PCR Kit (Qiagen) in a volume of 25 µL containing 12.5 µL of 2× PyroMark PCR Master Mix, 1.25 µL of each PCR primer (5 µM), 2.5 µL of 10× CoralLoad Concentrate, 6.5 µL high purity water and 1 µL of bisulfite-treated template DNA. The PCR cycling program for both primer sets was composed of an initial Taq activation/DNA denaturation step at 95 °C for 15 min, followed by 50 cycles of denaturation at 95 °C for 30 s, annealing at 58 °C for 30 s and elongation at 72 °C for 30 s. The program was finished by a final elongation step at 72 °C for 10 min. Seven microliters of PCR products were visualized by gel electrophoresis, and 10 µL were subjected to the sample preparation process for pyrosequencing. DNA was mixed with streptavidin-coated sepharose beads, followed by strand separation and washing utilizing the vacuum prep tool (Qiagen). The single-stranded DNA bound to the sepharose beads was mixed with 20 µL of 0.375 µM sequencing primer in annealing buffer and heated to 80 °C for 5 min. For the sequencing reaction PyroMark advanced reagents were used (Qiagen). The sequencing results were analyzed using the Advanced PyroMark software (Qiagen). A control PCR reaction without template DNA (no-template control) was included in the assay. PyroMark assays were carried out 2 times for accuracy.

### 4.5. Quantitative Real-Time PCR

Total RNA was isolated from biopsies and cells using the QIAGEN Allprep DNA/RNA Mini Kit (Qiagen, Hilden, Germany) and quantified with a NanoDrop ND-1000 (PeqLab, Erlangen, Germany). Two micrograms of RNA were treated with DNAse I (Amplification Grade, Invitrogen) at 37 °C for 30 min to eliminate possible DNA contamination and reverse transcribed by using the High Capacity cDNA Reverse Transcriptase Kit (Applied Biosystems). cDNA was diluted 1:10 with nuclease-free water. Quantitative analysis of specific mRNA expression was performed by real-time PCR on a LightCycler® 96 System (Roche, Laval, PQ, Canada). The 20-µL reaction mix contained 1.5 µL of 5 µM of each primer (please see the table for the primer sequences), 10 µL of SsoAdvanced™ Universal SYBR® Green Supermix (Bio-Rad, Mississauga, ON, Canada), 3 µL of nuclease-free water and 4 µL of diluted cDNA. No-template controls were run on each plate to test for the contamination of any assay reagents. The thermocycling conditions were initial denaturation at 95 °C for 5 min, followed by 45 cycles of denaturation at 95 °C for 10 s, annealing at 58 °C for 15 s and extension at 60 °C for 30 s. Melting curve analysis was performed by the end of each cycle to ascertain the specificity of the primers and the purity of the final PCR product. Assays were run in duplicate. The amount of target was calculated by the formula, 2^−(C^_t_^Gene- C^_t_^-GAPDH)^ × 1000, in which C_t_ is the threshold cycle value. All PCR results were carried out 3 times for accuracy. In addition, PPIA (peptidylprolyl isomerase A) was used to normalize our results as an independent normalization besides GAPDH [58].

### 4.6. Statistical Analysis

Regression and correlation analysis of average methylation percentage *versus* individual CpG methylation percentage was performed using GraphPad Prizm 5 software.

### 4.7. TCGA Database Analysis

Level 3 TCGA RNAseqV2 gene expression data were obtained from the TCGA Data Portal [59] in August 2014 for breast cancer (BRCA), colorectal adenocarcinoma (COAD), lung adenocarcinoma (LUAD) and pancreatic adenocarcinoma (PAAD). Correlations were tested between the gene (DNMT1, DNMT3B and MOAP1) and isoform (RASSF1A: uc003dea.1; and RASSF1C: uc003dab.1) expression values (log_2_ [RSEM normalized values+1]). A value of 1 was added to RSEM normalized values to avoid infinite values in log calculations. We conducted all analyses and visualizations in the RStudio programming environment (v0.98.501). R/Bioconductor packages ggplot2 and plyr were used where appropriate.

## 5. Conclusions

*RASSF1A* methylation analysis revealed the importance of investigating individual CpGs as average *RASSF1A* methylation could obscure the methylation hotspots and underestimate methylation percentage. In addition, we can observe a positive correlation between RASSF1A and MOAP-1 in breast, colorectal and thyroid cancers upon empirical testing by qPCR. Lastly, in breast, thyroid and colorectal cancers, the ratio of RASSF1A to RASSF1C changes in the malignant state to suggest caution when obtaining mRNA expression levels from microarray studies in genomic databases. 

## Figures and Tables

**Table 1:** Primer sequences for the pyrosequencing and qRT-PCR.

Assay		Primer Sequence 5’-3’
Pyrosequencing		
Assay 1 (20 CpGs)	Forward	GGATGTGGGGATTTTTTTTTTTTAGTATAG
	Reverse	Biotin-ACTTCAACCCCTCCCCCAAAA
	Sequencing	TTTTTTAGTATAGTAAAGTTGGT
Assay 2 (12 CpGs)	Forward	TTTCGGGTTTTATAGTTTTTGTATTTAGGT
	Reverse	Biotin-ACTCCCGCAACTCAATAAACTCAAACTC
	Sequencing	TTTAGTAGTTTAGTTTGGATTTTGG
MOAP1	Forward	GAGTGTTAGTTAGAGTTTAGGGGAGTTT
	Reverse	Biotin-CTCACCCCTCCCAACCCT
	Sequencing	AGGGGAGTTTGTTTT
qRT-PCR		
GAPDH	Forward	CATGACAACTTTGGTATCGTG
	Reverse	GTGTCGCTGTTGAAGTCAGA
RASSF1a	Forward	CCTCTGTGGCGACTTCATCTG
	Reverse	CAACAGTCCAGGCAGACGAG
MOAP-1	Forward	CTCAATTGCTCCTCTCTGTACC
	Reverse	CATGACAACTTTGGTATCGTG
RASSF1c	Forward	GCTACTGCAGCCAAGAGGAC
	Reverse	AGGTGTCTCCCACTCCACAG
PPIA	Forward	GCCGAGGAAAACCGTGTACT
	Reverse	TGTCTGCAAACAGCTCAAAGG

## Supplementary Materials

The following are available online at http://www.mdpi.com/2072-6694/8/6/55/s1, Figure S1: Correlation analysis of *RASSF1A* promoter methylation at the individual CpG site with average methylation percentage of 32 CpGs, Figure S2: *MOAP1* CpG island map and the pyromark assays, Figure S3: *RASSF1A* expression and methylation in colorectal cancer cell lines.
