# Supplementary material for: RASSF1A Site-Specific Methylation Hotspots in Cancer and Correlation with RASSF1C and MOAP-1
Source: Cancers (Basel). 2016 Jun 10;8(6):55. doi: 10.3390/cancers8060055 (PMC4931620; doi:10.3390/cancers8060055)
Supplement: Supplementary file 1 [file cancers-08-00055-s001.pdf]

RASSF1A Site-Specific Methylation Hotspots in Cancer and Correlation with *RASSF1C* and *MOAP-1*

Natalia Volodko, Mohamed Salla, Alaa Zare, El-Arbi Abulghasem, Krista Vincent, Matthew G.K. Benesch, Todd P.W. McMullen, Oliver F. Bathe, Lynne Postovit and Shairaz Baksh

(a) Correlation of Individual CpGs Methylation with Average Methylation of 32 CpGs in RASSF1a promoter

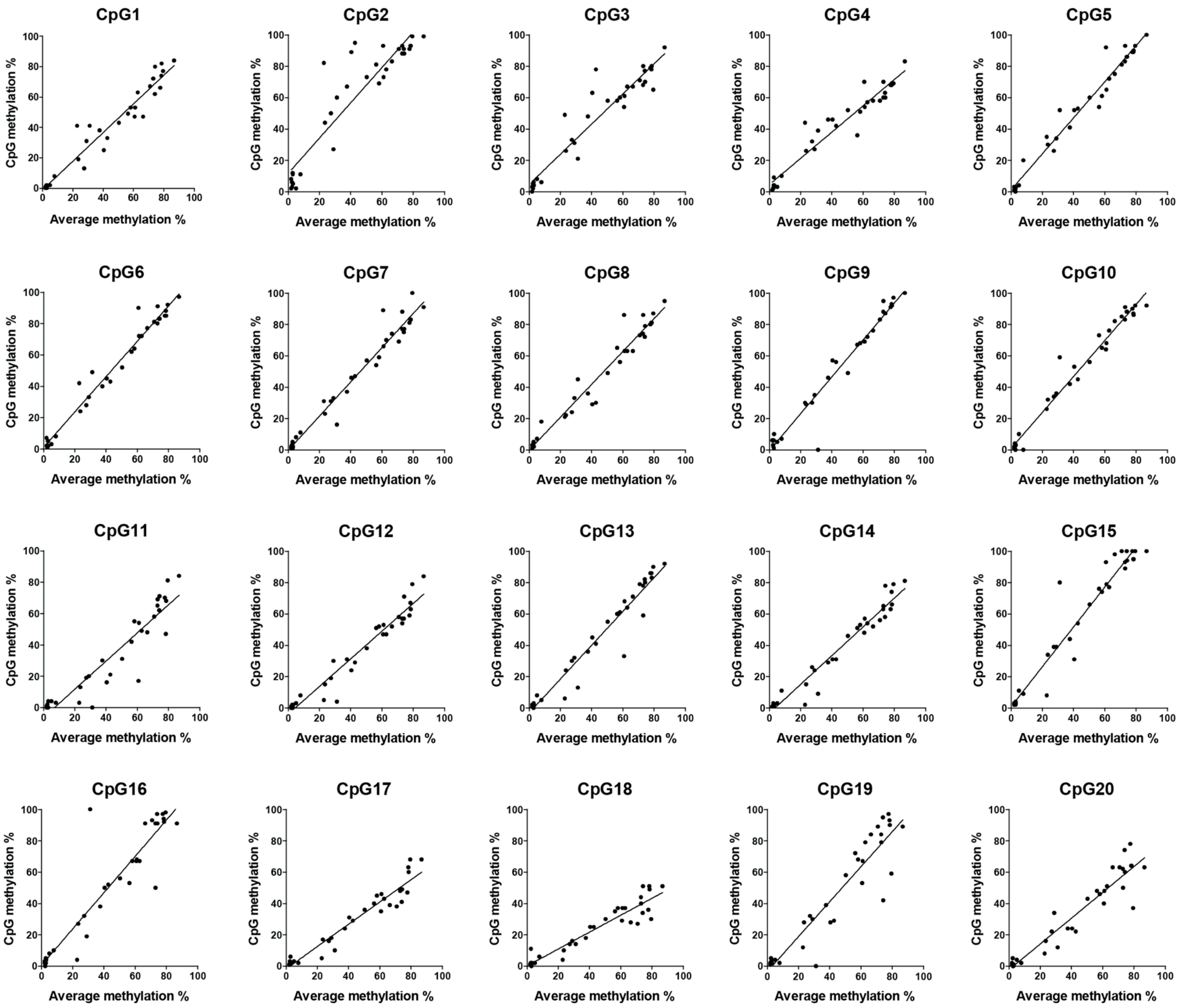

Figure S1. ***Cont.***

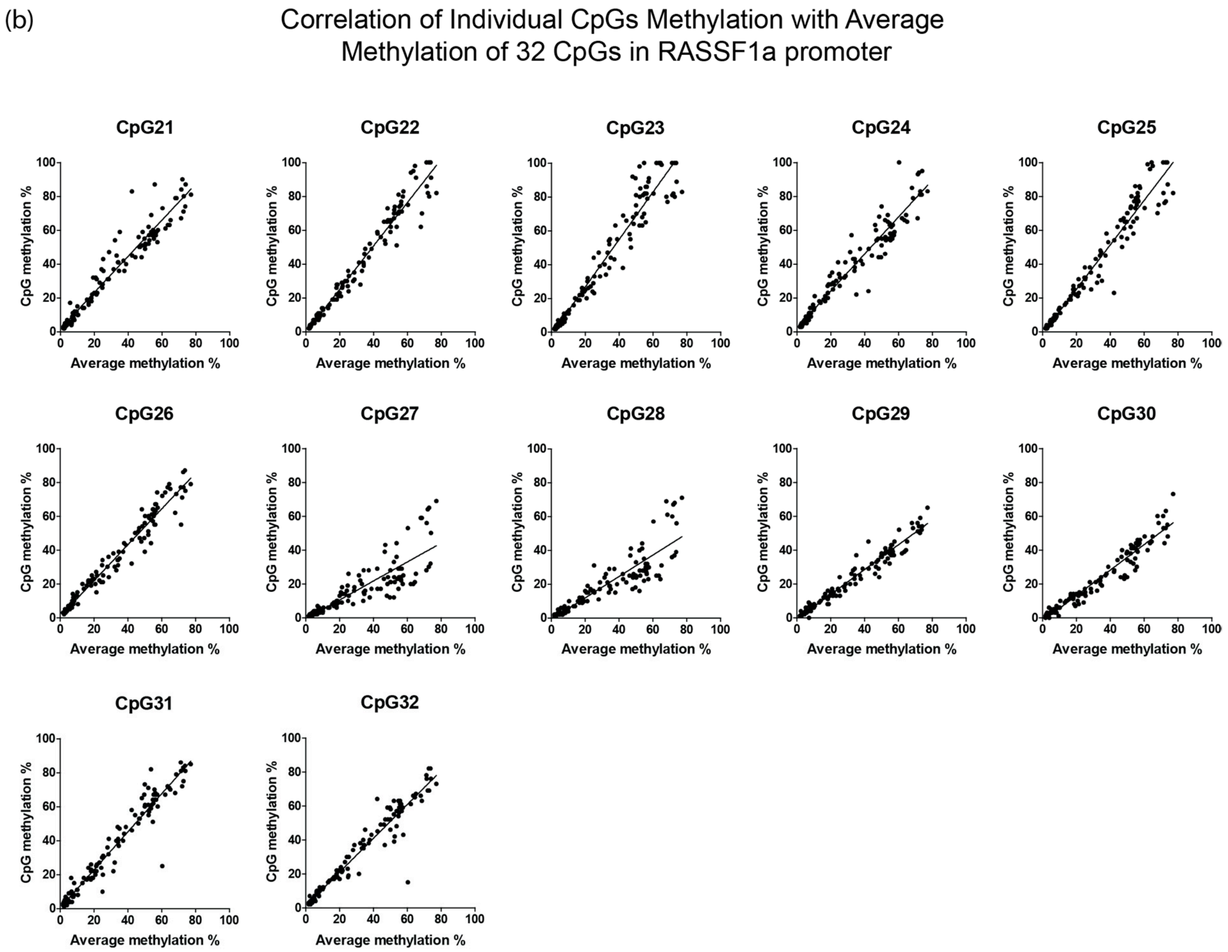

Figure S1. ***Cont.***

(c)

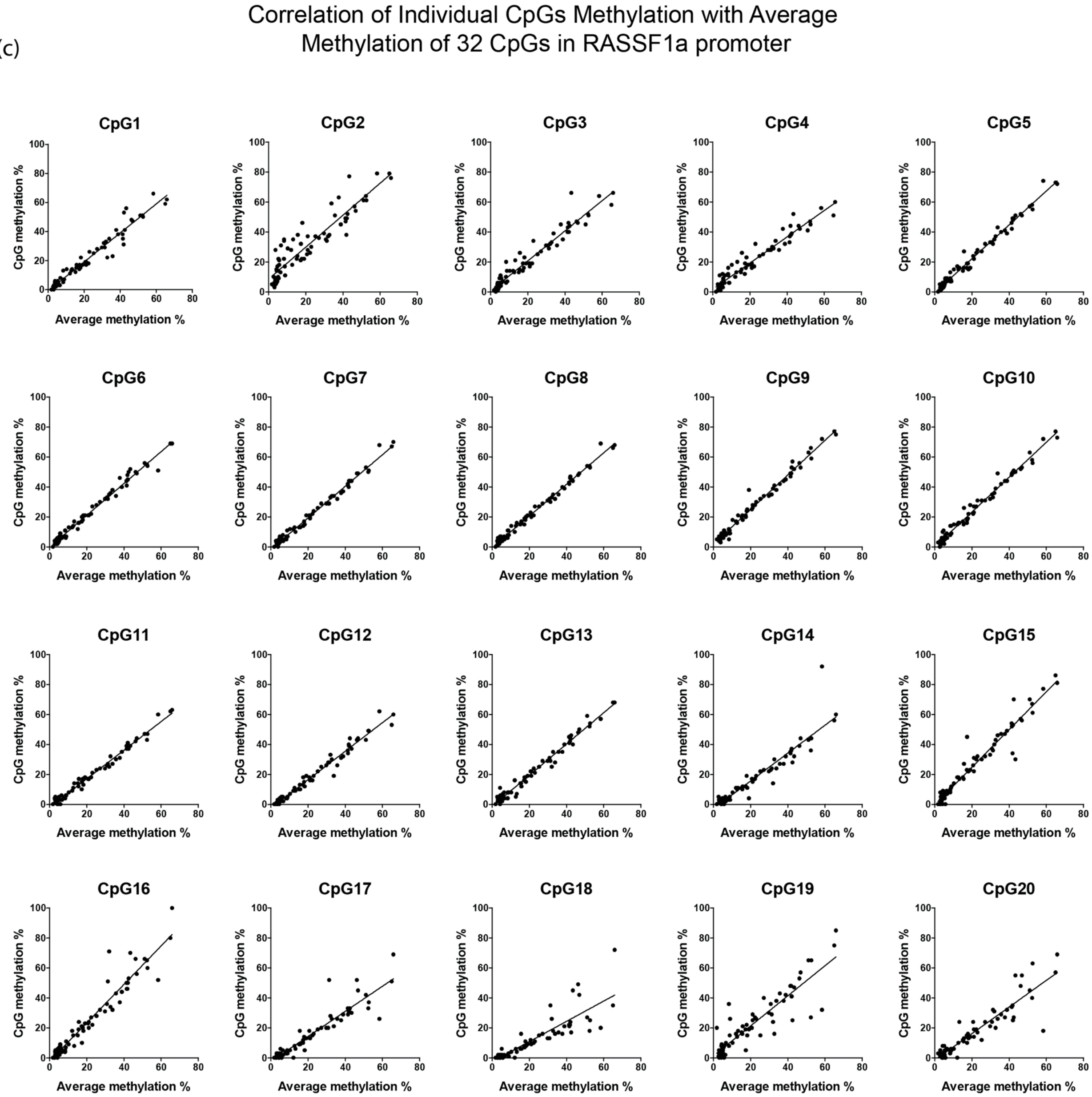

Figure S1. ***Cont.***

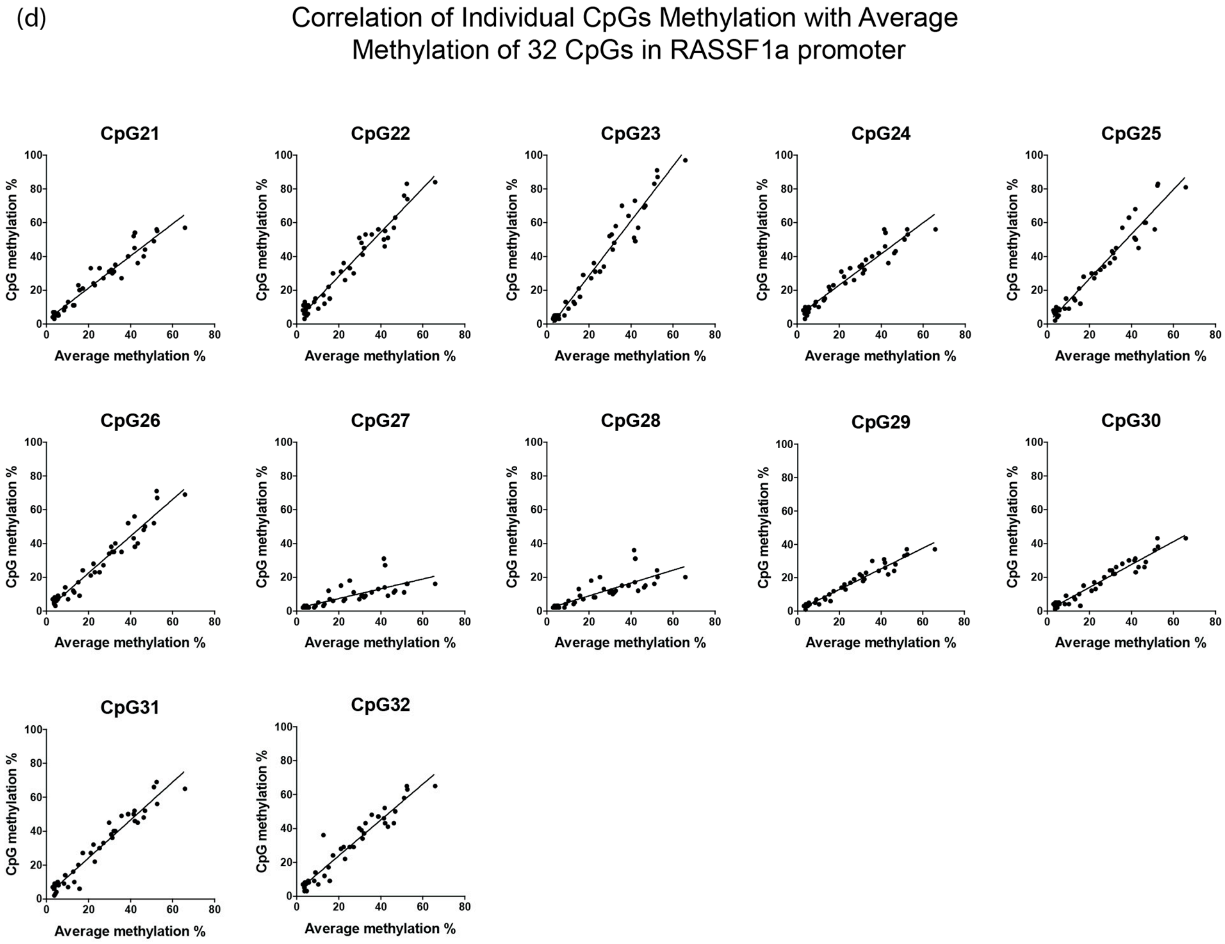

Figure S1. Correlation analysis of *RASSF1A* promoter methylation at the individual CpG site with average methylation % of 32 CpGs in cell lines **(a–b)** and breast cancer patients **(c–d).** The x-axis indicates average methylation of 32 CpGs in samples. The y-axis indicates individual CpG methylation percentage measured by pyrosequencing. Methylation of all CpGs correlated well with average methylation (r^2 values ranged between 0.7926 for CpG28 and 0.9939 for CpG9 for cell lines; and between 0.5777 for CpG27 and 0.9886 for Cpg7 for breast cancer patients; all *p* values are <0.0001).

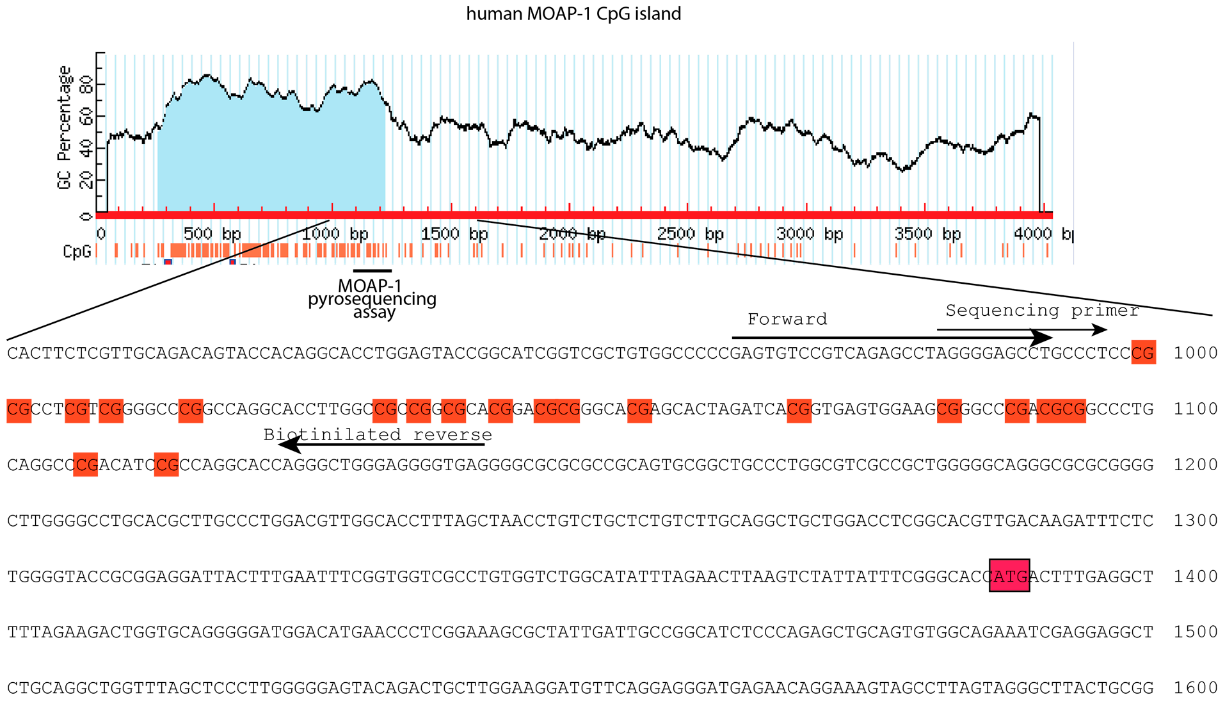

Figure S2. *MOAP1* CpG island map and the pyromark assays. *MOAP1* CpG island was predicted by Methprimer [19]. CpG sites are indicated by red strikes. Pyrosequencing assay is shown as black line. Start codon is indicated by an arrow. **(b)** *RASSF1A* bisulfite modified DNA sequence with studied CpGs and primers locations. Start codon is indicated by black rectangle. 20 CpGs interrogated by assay1 are highlighted in green, 12 CpG of assay 2 are highlighted in orange.

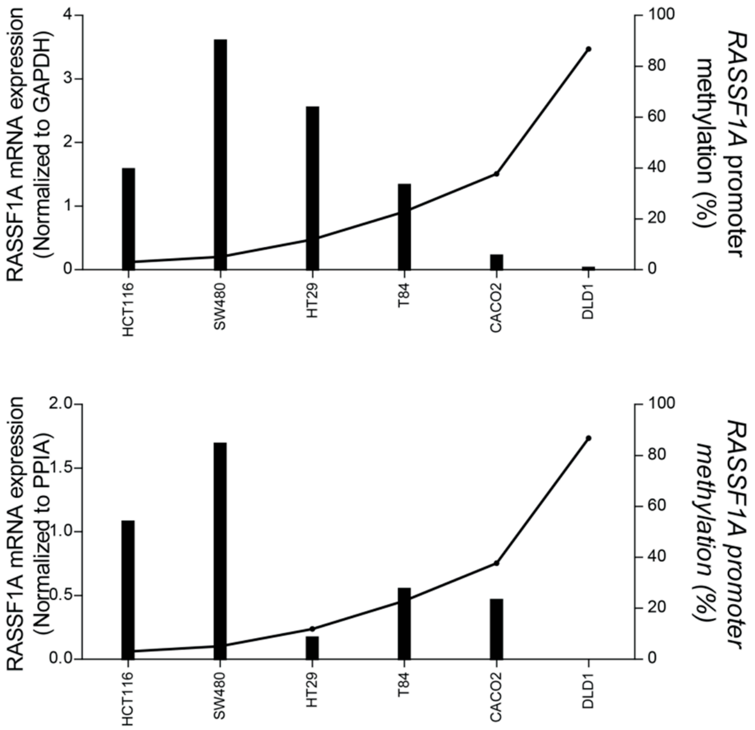

Figure S3. *RASSF1A* expression and methylation in colorectal cancer cell lines. *RASSF1A* methylation results in its mRNA expression silencing in cell lines. GAPDH **(a)** and peptidylprolyl isomerase A **(b)** mRNA expression were used independently to normalize *RASSF1A* expression.
